# Comparison of lipid profiles and inflammation in pre- and post-menopausal women with cerebral infarction and the role of atorvastatin in such populations

**DOI:** 10.1186/s12944-018-0669-9

**Published:** 2018-02-02

**Authors:** Jinhong Zhang, Hong Wang, Shuying Yang, Xiufen Wang

**Affiliations:** Department of Neurology, Cangzhou People’s Hospital, No.7 North Qingchi Road, Cangzhou, Hebei Province 061000 China

**Keywords:** Menopause, Atorvastatin, Ischemic stroke, Dyslipidemia, Systemic inflammation

## Abstract

**Background:**

The risks of atherosclerotic cardiovascular and cerebrovascular diseases in women rapidly increase with age in post-menopausal women. We aimed to investigate the lipid profiles in peri-menopausal women with cerebral infarction and to explore the effects of atorvastatin intervention.

**Methods:**

We collected women aged 40-60 with cerebral infarction between January 2013 and December 2016. Atorvastatin was applied for 6 months in all included patients. Blood lipid profiles, serum pro-inflammation cytokines, intracranial plaque and NIH stroke scale (NIHSS) scores were evaluated before and after atorvastatin treatment.

**Results:**

Totally 210 patients were included. Before atorvastatin treatment, post-menopausal patients had significantly higher levels of triglyceride, cholesterol, low-density lipoprotein and a reduced level of high-density lipoprotein than those in pre-menopausal patients. Blood levels of pro-inflammatory cytokines including interleukin (IL)-1, IL-6 and tumor necrosis factor-α were higher in post-menopausal patients, who had larger intracranial plaques than pre-menopausal patients. Consistently, post-menopausal patients had higher NIHSS scores than pre-menopausal ones. Atorvastatin reduced NIHSS scores and improved dyslipidemia in patients and eliminated the differences of these parameters between pre- and post-menopausal patients.

**Conclusions:**

Post-menopausal patients were severer than pre-menopausal patients in terms of dyslipidemia, systemic inflammation and NIHSS scores. Atorvastatin may be beneficial for women with cerebral infarction.

## Background

Atherosclerotic cardiovascular and cerebrovascular diseases are the most common causes of human death worldwide. Many risks have been identified, and among them, dyslipidemia, especially increased serum level of cholesterol is believed to be the most important one [1]. Hyperlipidemia causes atherosclerosis of vessels by inducing endothelial injury and smooth muscle cell proliferation via various ways [2]. Once feeding vessels of the heart and brain are involved, the risks of myocardial infarction and cerebral infarction, respectively, will greatly increase.

While hormones influence blood lipid profile, menopause can significantly alter women’s lipid profile, including those with potent atherogenic capacity. Epidemiologic studies have demonstrated the difference of lipid profiles between pre- and post-menopausal women, showing increased levels of low-density lipoprotein (LDL) and total cholesterol (TC), and a decreased level of high-density lipoprotein (HDL) [3, 4]. These menopause-related alterations of lipid profile were also proved to be associated with the rate of cardiovascular events, though contradictory results existed [5, 6]. The high incidence of stroke in elder women is partially contributed by the menopausal transition-related hormone changes [7]. Physiologically, estrogen can maintain a high level of HDL cholesterol and low levels of LDL cholesterol and triglycerides (TG) [8]. The dramatic reduced level of estrogen in post-menopausal women usually leads to dyslipidemia and is closely related to cardiovascular and cerebrovascular diseases [8, 9].

Statins, known as 3-hydroxy-3-methylglutaryl (HMG)-CoA reductase inhibitors, are a class of chemicals showing efficient lipid-lowering effect. While estrogen replacement was not recommended for the management of dyslipidemia in women, statin therapy could reduce the risks of cardiovascular events [1, 10]. Similarly, statin treatment was proved to prevent ischemic stroke [11]. For patients with cerebral infarction, statin treatment significantly reduced their morbidity and mortality. We thus hypothesized that statin could improve dyslipidemia in peri-menopausal women. Atorvastatin is a widely used statin and was reported to improve dyslipidemia in patients [12]. Consistently, the Stroke Prevention by Aggressive Reduction in Cholesterol Levels (SPARCL) trial showed that atorvastatin application significantly reduced incidence of recurrent stroke and fatal stroke [13, 14]. However, whether atorvastatin has different effects of stroke prevention in pre- and post-menopausal women is poorly understood. To figure this out, firstly we investigated the lipid profile and systemic inflammatory status in pre- and post-menopausal women with cerebral infarction. Then we explored the efficacy of atorvastatin in these patients to see whether it could reduce the risk of stroke by improving dyslipidemia.

## Methods

### Patients and atorvastatin treatment

We prospectively collected women patients diagnosed and treated in our department during the period from January 2013 to December 2016. The ages of recruited patients ranged from 40 to 60, and these patients were classified into pre-menopausal and post-menopausal groups according to their self-report. Our exclusion criteria were (1) refusal to participate in the study by patients themselves or their family members, (2) having severe diseases or the vital signs were unstable, (3) poor mental agility or incapable of communicating, (4) hepatic or renal insufficiency, (5) contraindication to statins, and (6) history of using lipid-lowering agents. The protocol of this study was reviewed and approved by the ethical committee of Cangzhou People’s Hospital. All patients provided written informed consent.

Atorvastatin was prescribed as 40 mg per day for all included patients regardless the menopause status. Participants were followed up after 0.5, 1, 3 and 6 months after atorvastatin treatment.

### Lipid profiling

Lipid profiling was performed as previously described [15]. Briefly, patients’ blood was collected and serum was isolated. Levels of TC and TG were determined by colorimetric methods (Kit Spinreact, Santa Coloma, Spain). Concentration of HDL-C was tested by precipitation with magnesium chloride and phosphotungstate (Kit Spinreact). LDL-C was calculated according to the previously published formula [16].

### Magnetic resonance angiography (MRA)

Intracranial atherosclerotic plaque was analyzed using MR [17]. We used time-of-flight MRA to assess the intracranial vasculature of our patients. All imaging was performed using a 1.5 Tesla system (Magnetom Sonata, Siemens, Erlangen).

### Stroke severity assessment

We used National Institutes of Health Stroke Scale (NIHSS) to quantify the stroke-caused impairment in our patients. All patients were assessed at indicated time according to the protocol of the National Institute of Neurological Disorders and Stroke [18].

### Enzyme-linked immunosorbent assays (ELISA)

Patients’ serum was collected by centrifugation at 600 g for 10 min at 4 °C, and was stored at − 80 °C until used. Levels of IL-1, IL-6 and TNF-α were determined using specific ELISA kits (all from R&D Systems, Minneapolis, MN) according to the manufacture’s instructions.

### Statistical analysis

Data were presented as the mean ± standard deviation (SD). All statistical analyses were conducted using unpaired two-tailed Student’s *t*-test. *P* values less than 0.05 were considered statistically significant.

## Results

### Lipid profiles and systemic inflammation of pre- and post-menopausal women with cerebral infarction

**Fig. 1:**
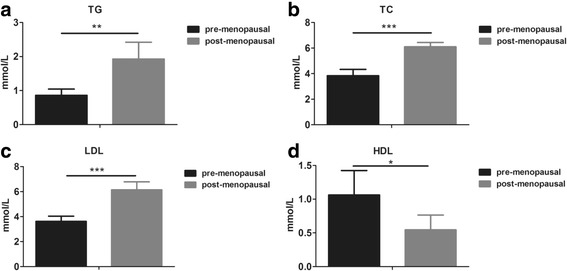
Comparison of lipid profiles in pre- and post-menopausal women with cerebral infarction. Serum levels of (**a**) TG, (**b**) TC, (**c**) LDL and (**d**) HDL were evaluated in pre- and post-menopausal women with cerebral infarction. TG, triglyceride; TC, total cholesterol; LDL, low-density lipoprotein; HDL, high-density lipoprotein. All values are presented as mean ± SD. *, *P* < 0.05; **, *P* < 0.01; ***, *P* < 0.001

**Fig. 2:**
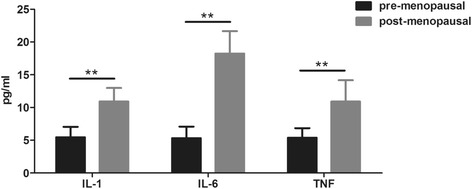
Serum levels of pro-inflammatory cytokines in pre- and post-menopausal women with cerebral infarction. Serum levels of IL-1, IL-6 and TNF in pre- and post-menopausal women were evaluated by Elisa assays. All values are presented as mean ± SD. **, *P* < 0.01

Totally 210 patients with acute cerebral infarction were included, with 102 pre-menopausal women and 108 post-menopausal women (Table 1). At baseline, the two groups showed significant difference in blood lipid profile. The levels of TG, TC, and LDL in the post-menopausal women were 2.2-fold, 1.6-fold, and 1.7-fold of those in the pre-menopausal patients (Fig. 1a-c). The HDL level, however, was decreased in post-menopausal women. We then evaluated the systemic inflammation in the two groups by testing three critical pro-inflammatory cytokines in the peripheral blood of patients. Post-menopausal women had much higher levels of IL-1, IL-6, and tumor necrosis factor (TNF)-α (Fig. 2). Since we have excluded patients with infectious diseases and other diagnosed chronic inflammatory diseases that would influence the levels of pro-inflammatory cytokines, the increased levels of pro-inflammatory cytokines were supposed to be menopause-related. Altogether, these results clearly showed severer dyslipidemia and systemic inflammation in the patients after menopause.

**Table 1:** Characteristics of the patients

Characteristics	Pre-menopausal (*n* = 102)	Post-menopausal (*n* = 108)
Age (years)	46.9 ± 5.3	54.4 ± 6.2
Weight (kg)	74.1 ± 12.8	71.8 ± 12.2
Height (m)	1.59 ± 0.06	1.58 ± 0.05
YSM (years)	–	5.8 ± 4.7
SBP (mmHg)	116 ± 13	132 ± 16**
DBP (mmHg)	80 ± 9	80 ± 10

*YSM* years since menopause, *SBP* systolic blood pressure, *DBP* diastolic blood pressure

Data are presented as means ± SD, p < 0.01**

### Intracranial plaque is lager in post-menopausal patients

**Fig. 3:**
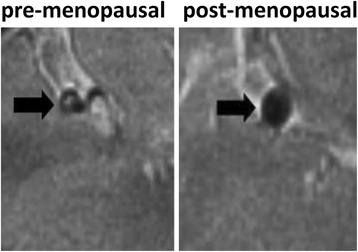
Comparison of Intracranial plaques in pre- and post-menopausal women with cerebral infarction. Intracranial plaque was larger in post-menopausal women compared with that in pre-menopausal women with cerebral infarction as revealed by MRA. Black arrows indicate intracranial plaques

TC is an independent risk factor of atherosclerosis in the carotid artery [19]. Hyperlipidemia may not only lead to an increased incidence of atherosclerosis, but may also influence the area of atherosclerotic plaque in the vessels. We therefore used magnetic resonance angiography (MRA) to assess the intracranial plaques in pre- and post-menopausal patients with cerebral infarction. It turned out that post-menopausal women had significantly larger plaque than pre-menopausal ones (Fig. 3), suggesting severer atherosclerosis in post-menopausal patients.

### Atorvastatin reduces NIHSS score in both pre- and post-menopausal patients

**Fig. 4:**
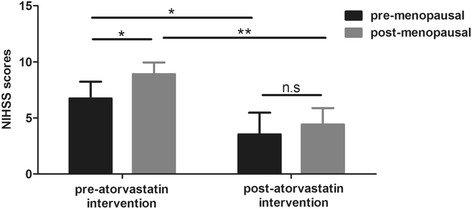
NIHSS scores in pre- and post-menopausal women with cerebral infarction before or after atorvastatin intervention. NIHSS scores of pre- and post-menopausal women with cerebral infarction were evaluated before and after 6 months of atorvastatin intervention. All values are presented as mean ± SD. *, *P* < 0.05; **, *P* < 0.01

We then tested the NIHSS scores in both groups. Post-menopausal women had higher scores than pre-menopausal patients at baseline (Fig. 4), suggesting that the disease of post-menopausal patients was generally severer when diagnosed. After 6-month treatment of atorvastatin, NIHSS scores in both groups were significantly lowered down, and the difference between the two groups was eliminated. These results implied that atorvastatin treatment seemed helpful for the recovery of cerebral infarction, and the clinical benefit in post-menopausal women was even larger.

### Atorvastatin removes difference in lipid profiles between pre- and post-menopausal patients

**Fig. 5:**
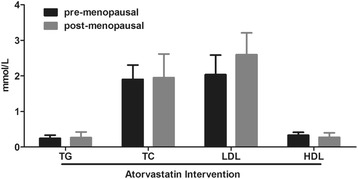
Lipid profiles in pre- and post-menopausal women with cerebral infarction after atorvastatin intervention. Serum levels of TG, TC, LDL and HDL were evaluated in pre- and post-menopausal women with cerebral infarction after 6 months of atorvastatin intervention. TG, triglyceride; TC, total cholesterol; LDL, low-density lipoprotein; HDL, high-density lipoprotein. All values are presented as mean ± SD

Since the use of atorvastatin improved NIHSS scores in patients, we than tested the lipid profiles after 6-month treatment of the agent. Consistent to the similar NIHSS scores between the two groups after treatment, the lipid profiles of the two groups were also similar (Fig. 5). Atorvastatin reduced levels of TG, TC and LDL in both pre- and post-menopausal patients and no differences were found between the two groups (Figs. 1 and 5). Intriguingly, atorvastatin did not increase HDL level in the meantime. It is thus suggested that atorvastatin was probably beneficial for patients with cerebral infarction by decreasing atherosclerosis-related lipids.

## Discussion

It is well known that post-menopausal women have a high risk of atherosclerotic cardiovascular diseases due to dyslipidemia, and statins are recommended for treatment of dyslipidemia and for prevention of dyslipidemia-related diseases including cerebral infarction. However, the lipid profile of cerebral infarction women aged around 50 years old is less frequently studied. We focused on this special population whose lipid profile was supposed to be influenced by menopause, and observed the efficacy of atorvastatin in these patients.

Consistent with previous studies [20, 21], post-menopausal women in our cohort showed severer dyslipidemia than pre-menopausal women. In parallel, severer stroke was generally observed in post-menopausal patients revealed by NIHSS scores. These results implied a positive correlation between the severity of dyslipidemia and the severity of stroke. Thus, improvement of dyslipidemia in peri-menopausal women may be able to alleviate the impairment caused by an ischemic stroke. Although we were currently unable to show whether atorvastatin reduced the incidence of ischemic stroke in peri-menopausal women, other investigators demonstrated that application of statins was associated with a decreased LDL-C level and a reduced risk of ischemic stroke [22]. Evidence from a systematic review also supported the use of statin in acute ischemic stroke and was associated with good clinical outcomes [23]. Our results confirmed the clinical benefits of atorvastatin in peri-menopasual women.

In addition to the up-regulated levels of TC, TG and LDL, we also observed significant increased levels of several pro-inflammatory cytokines in patients’ peripheral blood. The Women’s Health Initiative (WHI) Observational Study showed that C-reactive protein, another biomarker of systemic inflammation, was an independent predictor of ischemic stroke in post-menopausal women [24]. The systemic inflammation observed in post-menopausal women was believed to be gonadal steroids-related. However, the relationship between estrogen and inflammation can be complicated and probably bidirectional. On one hand, growing literatures suggest that a physiological level of estrogen is anti-inflammatory and estrogen deficiency after menopause appears pro-inflammatory [25]. On the other hand, exogenous administration of estradiol stimulated release of pro-inflammatory cytokines in animal models [26]. In agreement, the WHI clinical trial revealed that estrogen, with or without progestone, actually increased the risk of ischemic stroke in post-menopausal women [27]. Thus, estrogen supplement is not recommended for patients suffered from cerebral infarction. Although the mechanisms have not yet been fully elucidated, the bidirectional effects of estrogen in inflammation may explain the facts that pre-menopausal women have a low risk of stroke and that hormone replacement therapy fails to reduce the risk of ischemic stroke.

The high levels of TC and inflammatory cytokines in post-menopausal patients may be not coincident. Even without the influence of estrogen, chronic inflammation itself can decrease HDL-C and increase very-low-density lipoproteins (VLDL) [28]. While the two factors are both involved in stroke etiology and pathology [22, 29], statins show both lipid lowering effects and anti-inflammatory roles in atherogenesis [30]. The Heart Protection Study and SPARCL trial documented that both simvastatin and atorvastatin reduced recurrent stroke in patients with prior ischemic stroke [14, 31]. In addition, among several widely used statins, atorvastatin shows its best performance in reducing LDL-C at a dose of 40 mg/day and also seems the safest one at least in term of drug-related renal function [32]. In the present study, we used atorvastatin at a dose of 40 mg/day, and observed clinical benefits in peri-menopausal women patients. Noticeably, the effects of lipid lowering and NIHSS score reduction seemed correlated with each other in our study, suggesting that the benefits of symptom alleviation might be acquired by the improvement of dyslipidemia. Another interesting finding of our study is that atorvastatin eliminated the differences in lipid profiles and NIHSS scores between pre- and post-menopausal patients. This phenomenon indicated that though both groups acquired clinical benefits from atorvastatin administration, post-menopausal women appeared to have more because they had a more significant reduction of lipids. It may be possible that women patients with severer dyslipidemia can gain more clinical benefits from statin application. However, this speculation needs further validation because we currently do not know whether menopause participates in the phenomenon that atorvastatin shows enhanced lipid lowering in women patients with severer dyslipidemia.

In addition, our study suggested that the use of atorvastatin after cerebral infarction appeared to improve stroke severity in peri-menopausal women as reflected by reduced NIHSS scores. This result suggested that lipid lowering might be helpful to correct dysfunction of patients with cerebral infarction. Consistent with our findings, Mooins and co-workers proved that statins improved outcome in terms of NIHSS score and modified Rankin Scale in acute stroke patients [33]. However, some studies failed to show a role of statins in improving NIHSS scores [34]. This discrepancy reminds us that the clinical use of statins can be much more complicated. Many other factors such as the sample number, race, type and dosage of statins used, condition of patients, time of evaluation, and duration of treatment may influence the results. Thus, further studies are required to validate the clinical value of atorvastatin in peri-menopausal patients or even broader populations who had prior cerebral infarction.

Our study suggested that atorvastatin, or probably other statins, could improve dyslipidemia and be beneficial for functional outcome in peri-menopausal women. The use of statins in peri-menopausal women who suffered acute cerebral infarction is rarely reported. Although a recent systematic review and meta-analysis showed that statin therapy was associated with improved neurological outcome in patients [35]. However, peri-menopausal women are special because of their distinctive hormone alterations, which affect lipid profile and risk of stroke. We proved that atorvastatin worked in such population, and provided evidence for the clinical use of atorvastatin in peri-menopausal women with cerebral infarction.

## Conclusions

Compared to pre-menopausal women, post-menopausal women with prior cerebral infarction had severer dyslipidemia and systemic inflammation as well as severer impairment caused by stroke. Administration of atorvastatin improved dyslipidemia and NIHSS scores in peri-menopausal women.

## Abbreviations

HDL: High-density lipoprotein
IL: Interleukin
LDL: Low-density lipoprotein
NIHSS: National Institutes of Health stroke scale
TC: Total cholesterol
TG: Triglycerides
TNF: Tumor necrosis factor
